# Complex effects of environment and *Wolbachia* infections on the life history of *Drosophila melanogaster* hosts

**DOI:** 10.1111/jeb.14016

**Published:** 2022-05-09

**Authors:** Anton Strunov, Sina Lerch, Wolf U. Blanckenhorn, Wolfgang J. Miller, Martin Kapun

**Affiliations:** ^1^ Department of Evolutionary Biology and Environmental Studies University of Zurich Zurich Switzerland; ^2^ 27271 Department of Cell and Developmental Biology Center for Anatomy and Cell Biology Medical University of Vienna Wien Austria; ^3^ Natural History Museum of Vienna Wien Austria

**Keywords:** *Drosophila*, fitness, G × E interactions, temperature, *Wolbachia*

## Abstract

*Wolbachia* bacteria are common endosymbionts of many arthropods found in gonads and various somatic tissues. They manipulate host reproduction to enhance their transmission and confer complex effects on fitness‐related traits. Some of these effects can serve to increase the survival and transmission efficiency of *Wolbachia* in the host population. The *Wolbachia*–*Drosophila melanogaster* system represents a powerful model to study the evolutionary dynamics of host–microbe interactions and infections. Over the past decades, there has been a replacement of the ancestral *w*MelCS *Wolbachia* variant by the more recent *w*Mel variant in worldwide *D*. *melanogaster* populations, but the reasons remain unknown. To investigate how environmental change and genetic variation of the symbiont affect host developmental and adult life‐history traits, we compared effects of both *Wolbachia* variants and uninfected controls in wild‐caught *D*. *melanogaster* strains at three developmental temperatures. While *Wolbachia* did not influence any developmental life‐history traits, we found that both lifespan and fecundity of host females were increased without apparent fitness trade‐offs. Interestingly, *w*MelCS‐infected flies were more fecund than uninfected and *w*Mel‐infected flies. By contrast, males infected with *w*Mel died sooner, indicating sex‐specific effects of infection that are specific to the *Wolbachia* variant. Our study uncovered complex temperature‐specific effects of *Wolbachia* infections, which suggests that symbiont–host interactions in nature are strongly dependent on the genotypes of both partners and the thermal environment.

## Graphical abstract

The reasons for the recent global replacement of the Wolbachia wMelCS variant by wMel in Drosophila populations remain unknown. In our study, we investigated the fitness of wild‐caught D. melanogaster strains from Portugal at three developmental temperatures in presence and absence of these two Wolbachia types. The endosymbionts had no influence on life history traits that are determined during the development, such as development time, body size and number of ovarioles. In contrast, adult fitness components like oogenesis, fecundity and longevity were affected by temperature and Wolbachia type.

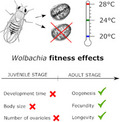

## 1 INTRODUCTION

The *Rickettsia*‐like Alphaproteobacteria *Wolbachia* are endosymbionts of many arthropod taxa that have profound effects on reproduction and other life‐history traits of the host (reviewed in Kaur et al., 2021; Landmann, 2019). They are transmitted vertically through the mother's egg to offspring and have evolved strategies that benefit females in the host population, thereby enhancing their own transmission (Werren et al., 2008). The microbe can manipulate the host reproductive system via different strategies like feminization (Rousset et al., 1992), parthenogenesis (Huigens et al., 2000), male killing (Jiggins et al., 2000) or cytoplasmic incompatibility (CI; Caspari & Watson, 1959), which altogether can result in up to 100% infected individuals in some species (Landmann, 2019). *Wolbachia* symbionts are prevalent and persistent in nature (Bykov et al., 2019; Solignac et al., 1994; Verspoor & Haddrill, 2011) as well as in established long‐term lab strains of the fruit fly *Drosophila melanogaster* (Clark et al., 2005). They usually do not induce any significant cytoplasmic incompatibility (CI) in natural populations (Bourtzis et al., 1996; Clark et al., 2002; Merçot & Charlat, 2004; Poinsot et al., 1998; Reynolds & Hoffmann, 2002), except under certain artificial laboratory conditions, for example, when males develop fast and are influenced by pheromones of their sister during metamorphosis (Pontier & Schweisguth, 2015; Yamada et al., 2007), or when paternal grandmothers are maintained as virgins for a long time before insemination (Layton et al., 2019). Several studies on fruit flies documented potential fitness benefits of *Wolbachia* infections in the form of higher fecundity or increased longevity (Fry et al., 2004; Fry & Rand, 2002; Olsen et al., 2001), while other studies showed no or even negative fitness consequences of infections (Harcombe & Hoffmann, 2004). Moreover, *Wolbachia* infections can induce resistance against RNA viruses in *D*. *melanogaster* (Hedges et al., 2008; Teixeira et al., 2008) and other *Drosophila* hosts (Martinez et al., 2014). Together, the mutualistic effects of *Wolbachia* in *D*. *melanogaster* may explain its pervasiveness in natural fly populations (Teixeira et al., 2008). However, when considering RNA virus protection, it is important to account for differences between laboratory populations and flies collected in nature (Cogni et al., 2021; Shi et al., 2018).

There are two bacterial variants that are most commonly found in natural *D*. *melanogaster* populations, named *w*Mel and *w*MelCS (Riegler et al., 2005), which diverged approximately 80 000 fly generations (Chrostek et al., 2013; Early & Clark, 2013; Richardson et al., 2012), that is, approximately 5.300 years ago (assuming 15 generations per year; Pool, 2015). Genetic analyses of long‐term lab strains and recently collected samples indicate a worldwide replacement of the ancestral *w*MelCS by the more recent *w*Mel variant within a few decades (Riegler et al., 2005). The reasons for this global turnover remain unknown. Since CI is generally weak in natural *D*. *melanogaster* populations (Hoffmann et al., 1994), differential fitness effects imposed by the two variants are the most plausible cause for this recent global turnover.

In *D*. *melanogaster*, fitness effects can differ with respect to *Wolbachia* variants (Chrostek & Teixeira, 2015). Fly strains infected with the *w*MelCS variant exhibit higher resistance against RNA viruses than strains infected with the closely related *w*Mel variant (Chrostek et al., 2013). This phenotypic difference may be caused by an overall higher titre of *w*MelCS (Chrostek et al., 2013): elevated titres may impose fitness costs on infected flies that result in a shorter lifespan due to competition for cellular resources (Chrostek et al., 2013). In its most extreme form, the *w*MelPop variant, a lab‐generated subvariant of *w*MelCS, induces the highest infection titre and significantly reduces lifespan at higher temperatures (Min & Benzer, 1997; Strunov et al., 2013).

Most of the aforementioned studies focused on intrinsic factors, such as different *Wolbachia* or host genotypes, which may influence the homoeostatic host–symbiont interactions. Much less is known about the influence of extrinsic environmental factors, such as temperature, nutrition or population density (Christensen et al., 2019; Hoffmann et al., 1998; Pontier & Schweisguth, 2015; Ponton et al., 2015; Reynolds et al., 2003). Recent studies indicate that individuals infected with *w*MelCS prefer cooler temperatures than uninfected individuals (Truitt et al., 2019; Arnold et al., 2019; but see Hague et al., 2020), suggesting that *Wolbachia*‐infected hosts may avoid higher temperatures to alleviate fitness costs resulting from bacterial infections. A new study of natural *D*. *melanogaster* populations from Ukraine infected with *w*Mel and *w*MelCS found that the effect of bacteria on fitness components and stress‐related phenotypes is highly condition‐dependent and influenced by the host genotype (Serga et al., 2021). However, most work focusing on phenotypic effects of *Wolbachia* infections used highly inbred, long‐term *Drosophila* lab strains, which were often generated by *de novo* introgression via backcrossing (Chrostek et al., 2013; Teixeira et al., 2008) or transinfection with non‐native *Wolbachia* variants (Martinez et al., 2014). Mutation accumulation, genetic drift and/or lab adaptation may all confound interactions between host and symbionts under laboratory conditions, thereby potentially leading to wrong conclusions about evolutionary mechanisms influencing the coexistence of *Wolbachia* and *Drosophila* in nature. To account for this, here we analysed various fitness‐related traits at different rearing temperatures and life stages in two wild‐caught *D*. *melanogaster* strains infected with either *w*Mel (RP1) or *w*MelCS (RP2) that were recently (2018) collected in Portugal. Given the current dominance of *w*Mel, we hypothesized that this variant imposes lower costs on host fitness than the ancestral *w*MelCS.

## 2 MATERIALS AND METHODS

### 2.1 Fly strains and husbandry

We worked with three presumably highly inbred isofemale *Drosophila melanogaster* strains that have been established from single gravid females that were collected in October 2018 from the wild in Recarei/Portugal. The first strain (RP1) carries the *w*Mel *Wolbachia* variant, the second (RP2) *w*MelCS and third (RP3) is naturally uninfected, as confirmed by PCR (see below). All experimental strains were maintained in incubators at 24°C, ca. 60% humidity, and a 12 h:12 h light/dark cycle prior to experiments.

### 2.2 Crosses among isofemale fly strains from Portugal

Phenotypic variation among the two strains with *w*Mel (RP1) and *w*MelCS (RP2) infections from Portugal may be a result of differences in *Wolbachia* infection types and/or their nuclear genetic background. In addition, high levels of inbreeding in the isofemale strains may result in inbreeding depression, which may negatively affect fitness. To homogenize the autosomal genetic background of RP1 and RP2, we took advantage of the strict maternal transmission of *Wolbachia* to obtain hybrid F1 offspring infected with either *w*Mel (derived from the *w*Mel female × *w*MelCS male cross) or *w*MelCS (from the *w*MelCS female × *w*Mel male cross). We further eliminated *Wolbachia* infections in the natural strains by treating flies from a subset of each strain with antibiotic (0.1% Rifampicin) for three generations and subsequently restored their gut flora (GFR) by placing the flies into food vials with freshly deposited fly faeces from untreated males of the same strain for two generations. We then used these antibiotic‐treated *Wolbachia*‐free strains to set up F1 flies similar to the crosses above (*w*Mel female GFR × *w*MelCS male GFR and *w*MelCS female GFR × *w*Mel male GFR) to compare flies with homogeneous genetic background in the presence or absence of *Wolbachia* infections.

Importantly, the direction of the cross may additionally influence phenotypic effects independently of *Wolbachia* infections. For example, males have only one (hemizygous) copy of the X‐chromosome, which they inherit from their mothers. Moreover, mitochondria are only transmitted maternally and thus differ in the F1 with respect to the direction of the cross. To account for this statistically, we decomposed the four possible *Wolbachia* infection outcomes from the crosses described above (*w*Mel+, *w*Mel−, *w*MelCS+, *w*MelCS−) into two main factors *cross* and *infection* in our statistical analyses. *Cross* is a factor with two levels (*w*Mel and *w*MelCS) which describe the direction of the crosses among the two pure isofemale strains, that is, RP1 × RP2 (which we denote *w*Mel) and RP2 × RP1 (which we denote *w*MelCS), irrespective of their infection status. This factor thus accounts for differences with respect to the direction of the cross among the two strains independently of the *Wolbachia* infection status, which may stem from maternally transmitted mtDNA or host sex‐linked effects. Conversely, the factor *infection* is a fixed factor with two levels (+, −), which describes the presence or absence of *Wolbachia* infections irrespective of the *Wolbachia* type. Thus, only a significant interaction between *cross* and *infection* indicates different effects of the two *Wolbachia* variants on the investigated phenotype.

### 2.3 Confirmation of *Wolbachia* infection with polymerase chain reaction (PCR)

To confirm the infection status of all experimental strains, we used PCR with *Wolbachia*‐specific primers and conditions as described by Riegler et al. (2005). In brief, we extracted genomic DNA of five pooled flies using the Qiagen DNeasy kit (Qiagen, Hilden, Germany) and amplified a sequence of the *wsp* gene, a well‐established maker for *Wolbachia* infections. Upon positive results for all infected strains for the *wsp* locus, we further amplified a variable tandem repeat region of the *Wolbachia* genome (VNTR‐141) which is characterized by length polymorphisms that are diagnostic for different *Wolbachia* variants (Riegler et al., 2012). PCR amplification was set up in 10 µl reaction volumes with 0.3 μM primers in 1× reaction buffer (Promega 5x Green GoTaq), 2.5 mM MgCl_2_, 150 µM dNTPs and 0.025 U/µl DNA‐Polymerase (Promega GoTaq). Following the protocol, we ran PCR reactions with the following conditions: 2 min at 94°C for initial denaturation followed by 30 cycles of 45 s at 94°C (denaturation), 45s at 67°C (annealing) and 30s at 72°C (elongation). The run finished with a final extension at 72°C for 10 min. To quantify length polymorphisms, we visualized and separated PCR bands by gel electrophoresis using a 0.8% agarose gel (see supplementary data file for gel images).

### 2.4 Development time

We measured egg to adult development time by counting eclosed F1 hybrids from all crosses described above (*w*Mel × *w*MelCS, *w*MelCS × *w*Mel, *w*MelCS GFR × *w*Mel GFR, *w*Mel GFR × *w*MelCS GFR). For each of the four crosses and each temperature regime (20°C, 24°C, 28°C), we allowed 15 individual females at an age of 4–7 days to lay eggs in individual vials (i.e. one female/vial) in one 24‐h interval. These vials were then placed into incubators at the respective temperatures. To measure development time, each vial was checked for newly eclosed adults three times per day (8:00, 14:00, 20:00) for 1 week starting from the day the first flies eclosed. Flies were sexed and subsequently used for a longevity experiment (see below).

### 2.5 Body size

Femur length is a reliable proxy for adult body size in *Drosophila* (Siomava et al., 2016), a fitness‐related adult trait that is determined during larval development, which is positively correlated with female fecundity (e.g. see Flatt, 2020), To investigate if *Wolbachia* influences female body size, we dissected and mounted the left foreleg of the F1 females emerging from all crosses described above on glass slides with Euparal mounting medium (Roth), to be photographed at 40x magnification using a Leica DFC490 digital camera attached to a Leica MZ12 microscope to estimate femur length with ImageJ (http://imagej.nih.gov/ij/; v.1.53c) based on two landmarks (as described in Debat et al., 2011), in triplicate to minimize measurement error (see Supplementary data file). Raw images of female forelegs can be obtained from DataDryad (https://doi.org/10.5061/dryad.sxksn035v).

### 2.6 Oogenesis and ovariole number

To obtain insight into the developmental mechanisms conferring fecundity differences between *w*Mel and *w*MelCS variants, we compared the number of ovarioles in females at different temperatures. We also counted the number of mature eggs in the ovarioles 24 and 48 h after eclosion. From each cross, we collected 20 freshly eclosed virgin females and paired each with two uninfected (RP3) males in incubators at 20, 24 and 28°C. To count ovarioles, we dissected female abdomens in phosphate‐buffered saline (PBS) and stained ovaries for ca. 5 min in potassium dichromate solution. Ovarioles and mature eggs in each ovariole were counted by eye at 16‐fold magnification under a Leica MZ12 microscope.

### 2.7 Fecundity

To assess the effect of *Wolbachia* variant and infection type on female fecundity, we used F1 virgin females that were generated from the four crosses described above (*w*Mel × *w*MelCS, *w*MelCS × *w*Mel, *w*MelCS GFR × *w*Mel GFR, *w*Mel GFR × *w*MelCS GFR). We paired single F1 virgin females (within 24h post eclosion) with two naturally uninfected males of strain RP3 and propagated these trios at 20, 24 or 28°C. For each temperature, cross and infection status, we set up 30 replicate triads to investigate their fecundity for 10 consecutive days. Every day, all flies were transferred to a new vial, and their offspring was counted every second day as the number of eclosing adults. Males that died during this time were not replaced.

### 2.8 Longevity

We assessed strain‐ and environment‐specific lifespans for each cross (described above), infection‐type and temperature. For each of three replicates per treatment combination, we collected 25 F1 females and males and placed them in 1 L demography cages at three temperatures (20, 24, 28°C). Plastic longevity cages had metal grid windows to ensure constant airflow and an opening permitting easy replacement of attached food vials in 48‐h intervals without releasing flies. This set‐up guarantees *ad libitum* food and prevents flies from drowning in the medium. Dead flies were collected, recorded and sexed every 24 h until all experimental flies had died. Flies that drowned in the food media or escaped were not included in the analysis.

### 2.9 Statistical analyses

We used *R* (version 4.0.3; R Core Team, 2019) to carry out all statistical analyses in Rstudio (version 1.4.1103; RStudio Team, 2020). Figures were created with the *ggplot2 R* package (Wickham, 2016). We analysed all outcome variables (listed above and below) as a fully crossed factorial design with three main fixed factors (*temperature*, *cross*, *infection*), including all possible higher order interactions, and *replicate trial* as random factor (only for the outcome variables development time, body size, ovariole number, mature eggs and longevity). *Temperature* was a (fixed) factor with three levels (20, 24, 28°C), *cross* a factor with two levels (*w*Mel and *w*MelCS, see above for a more detailed description) and *infection* a factor with two levels denoting the presence (+) versus absence (−) of *Wolbachia* infection. Additionally, for some outcome variables, *sex* was added as a fixed factor with two levels (male and female; for development time and longevity only), and *age* a fixed factor with two levels (24 and 48 h after adult eclosion) in case of ovariole number, and with five levels (1, 3, 5, 7, 9 days after eclosion) in case of fecundity (detailed below).

Body size at emergence was analysed with a regular general linear mixed model (LMM; including *temperature*, *cross*, *infection*) and normal error distribution and ovariole number with a generalized linear mixed model (GLMM; including *temperature*, *cross*, *infection*, *age*) with Poisson‐distributed errors to account for the statistical properties of count data, using the *R* package *lme4* (Bates et al., 2015). Development time and longevity (i.e. age at death) were analysed using proportional hazards (Cox regression) analysis with the *R* package *coxme* (Therneau, 2020; including *temperature*, *cross*, *infection*, *sex*). When scoring fecundity of females, we unfortunately not fully tracked the identity of individual females over time (female age when laying: 1, 3, 5, 7 and 9 days post eclosion). It was thus not possible to conduct the appropriate repeated‐measures analysis with female ID as random factor. To avoid inflated type‐II errors (i.e. falsely rejecting the null hypothesis) caused by the dependence of females across consecutive days, we did not enter female age as an additional factor in our analysis but analysed each day separately using general linear models (GLM; including *temperature*, *cross*, *infection*, *age*) with Poisson‐distributed errors to account for the statistical properties of count data. Due to an excess of females that did not produce offspring on day 1 (75.5%), this first time point was excluded from further statistical analyses to avoid excessive zero inflation. To further account for the non‐independence of the data across the four time points, we conservatively applied Bonferroni correction for multiple testing, that is, we only considered an effect significant if the *p*‐value was smaller than the corrected significance threshold α' = 0.0125 (0.05/4). Similarly, for the number of mature eggs in ovarioles, we investigated the two time points of the assay (24 and 48 h post‐eclosion) separately. Since most females at 24 h had not developed mature eggs (only 18% = 52 females carried one or more mature eggs), we only describe this subset qualitatively. We statistically analysed only the data of 48‐h‐old females (when ca. 82% = 239 of all females carried at least one mature egg) with a GLMM (including *temperature*, *cross*, *infection*) and a negative binomial error structure as implemented in the *R* package *glmmTMB* (Brooks et al., 2017) to account for zero inflation in the data set.

For GLMMs and cox regression, we tested for significance with type‐III analysis of deviance based on Wald *χ* ^2^‐tests as implemented in the *R* package *car* (Fox & Weisberg, 2019). For LMM and GLMs, we applied Satterthwaite's method to estimate degrees of freedom and tested for significance with Kenward–Roger *F*‐tests using the *car* package (Fox & Weisberg, 2019). We performed pairwise comparisons among levels for significant response variables with more than two factor levels using Tukey's honest significant difference (HSD) post hoc tests as implemented in the *R* package *emmeans* (Lenth, 2021). All raw data, *R* scripts with complete models, the code for statistical analyses and the original output files are provided as a zipped Supplemental Data file.

## 3 RESULTS

### 3.1 Development time

**FIGURE 1:**
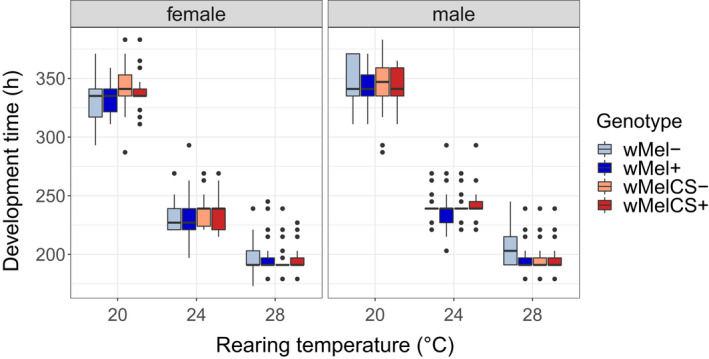
Development time of wild‐caught *D*. *melanogaster* from Portugal naturally infected with *w*Mel+ (blue) and *w*MelCS+ (red) *Wolbachia* variants at 20, 24 and 28°C in comparison to flies of the same strains that were treated with antibiotics: *w*Mel− (light blue) and *w*MelCS− (light red). Total *n* = 3946

Neither *Wolbachia infection* nor direction of the *cross* influenced the development time of the host (*Infection*: *χ* ^2^ = 0.2, *p* = 0.63 and *Cross*: *χ* ^2^ = 0.28, *p* = 0.6; Table 1; Figure 1 and Figure S1). However, we found significant effects of *temperature* and *sex* on development time in all strains (Table 1). Females eclosed before males (*Sex*; *χ* ^2^ = 208.2, *p* < 2.2e‐16) and developed faster at higher temperatures (*Temp*; *χ* ^2^ = 1650.9, *p* < 2.2e‐16; Figure 1, Table 1). Accordingly, we found highly significant interactions between *temperature* and sex (*χ* ^2^ = 36.4, *p* = 1.3e‐08). In addition, there was a significant interaction between *sex* and *cross* (*χ* ^2^ = 5.1, *p* = 0.024), which hints at sex‐linked effects on development time determined by the direction of the cross. Lastly, there was a marginally significant higher order interaction between *sex*, *temperature* and *infection* (*χ* ^2^ = 6.2, *p* = 0.044), suggesting very subtle differential effects of *Wolbachia* infections at different temperature and the sexes.

**TABLE 1:** Type‐III analysis of deviance testing for significant effects of *Wolbachia* infection, *Wolbachia* variant (i.e. direction of crosses), rearing temperature and sex on development time in *D*. *melanogaster*

Effect	*χ*2	*df*	*p*‐Value
**Sex**	**208.2**	**1**	**<2.2E‐16**
**Temp**	**1650.9**	**2**	**<2.2E‐16**
Cross	0.4	1	0.517
Infection	2.8	1	0.095
**Sex:Temp**	**36.4**	**2**	**1.3E‐08**
**Sex:Cross**	**5.1**	**1**	**0.024**
Temp:Cross	1.7	2	0.420
Sex:Infection	1.9	1	0.171
Temp:Infection	1.9	2	0.396
Cross:Infection	0.1	1	0.810
Sex:Temp:Cross	2.3	2	0.323
**Sex:Temp:Infection**	**6.2**	**2**	**0.044**
Sex:Cross:Infection	2.0	1	0.157
Temp:Cross:Infection	1.2	2	0.544
Sex:Temp:Cross:Infection	0.4	2	0.823

Note

Significant results are highlighted in bold.

### 3.2 Body size

**FIGURE 2:**
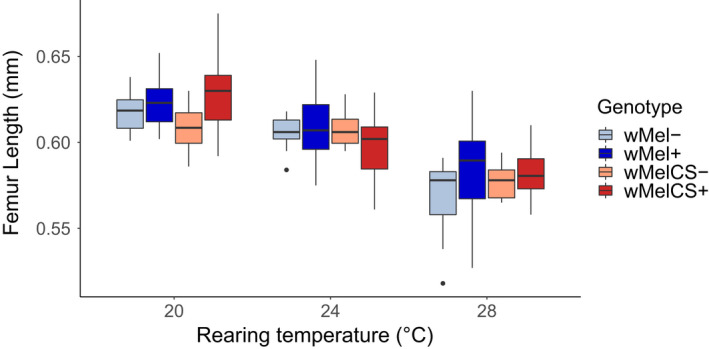
The left femur length of the first leg pair from *D*. *melanogaster* adult females (10 days post eclosion) naturally infected with *w*Mel+ (blue) and *w*MelCS+ (red) *Wolbachia* variants reared at 20, 24 and 28°C in comparison to flies of the same strain treated with antibiotics (*w*Mel−, light blue and *w*MelCS−, light red). Total *n* = 256

We investigated the potential influence of *Wolbachia* infections and developmental temperature on body size, which is determined during larval development. Fore femur length, a reliable proxy for adult body size (Siomava et al., 2016) was neither affected by *Wolbachia infection* (*F*_1,43.6_ = 3.0, *p* = 0.089, Table 2, Figure S2) nor by the direction of the *cross* (*F*_2,43.3_ = 0.1, *p* = 0.79, Table 2, Figure 2). By contrast, higher temperatures significantly decreased fore femur lengths (*F*_1,43.6_ = 34.7, *p* = 1.0e‐09).

**TABLE 2:** Type‐III ANOVA with Satterthwaite's method to approximate degrees of freedom testing for significant effects of *rearing temperature*, *Wolbachia infection* and *Wolbachia type* (i.e. direction of the *cross*) on femur length of female flies

Effect	*F*‐value	*df*	*p*‐Value
**Temp**	**34.7**	**1/43.6**	**1.0E‐09**
Cross	0.1	2/43.3	0.787
Infection	3	1/43.6	0.089
Temp:Cross	0.5	1/43.6	0.617
Temp:Infection	1.4	2/43.3	0.256
Cross:Infection	0.4	2/43.3	0.505
Temp:Cross:Infection	1.2	1/43.6	0.322

Note

The column ‘*df*’ shows degrees of freedom of numerator and denominator separated by a dash. Significant results are highlighted in bold.

### 3.3 Ovariole number

**FIGURE 3:**
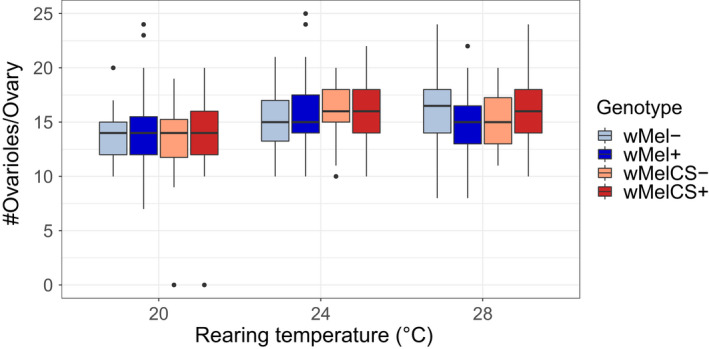
Mean number of ovarioles in adult females (1–2 days old) with different *Wolbachia infections* (*w*Mel+: blue and *w*MelCS+: red; light colours when treated with antibiotics [−]). Total *n* = 588

We further tested if differences in larval development potentially affect female fecundity via the number of ovarioles. While *temperature* had a significantly positive effect on the total number of ovarioles (*X* ^2^ = 27.2, *p* = 1.3e‐06, Table 3, Figure 3), we neither found an effect of *Wolbachia infection* (*X* ^2^ = 0.5, *p* = 0.474) nor differences with respect to direction of the *crosses* (*X* ^2^ = 0.9, *p* = 0.335) nor *age* of the female (*X* ^2^ = 2.8, *p* = 0.096).

**TABLE 3:** Type‐III analysis of deviance testing the effects of rearing *temperature*, *infection* status and *Wolbachia* type (i.e. direction of the *cross*) on number of ovarioles from one female

Effect	*χ* ^2^	*df*	*p*‐Value
Age	2.8	1	0.096
**Temp**	**27.2**	**2**	**1.3E‐06**
Cross	0.9	1	0.335
Infection	0.5	1	0.474
Age:Temp	0.2	2	0.913
Age:Cross	2.0	1	0.160
Temp:Cross	1.0	2	0.600
Age:Infection	0.2	1	0.692
Temp:Infection	0.4	2	0.802
Cross:Infection	0.8	1	0.381
Age:Temp:Cross	5.5	2	0.065
Age:Temp:Infection	0.8	2	0.671
Age:Cross:Infection	0.4	1	0.547
Temp:Cross:Infection	1.5	2	0.463
Age:Temp:Cross:Infection	0.2	2	0.895

Note

Significant results are highlighted in bold.

### 3.4 Female fecundity

**FIGURE 4:**
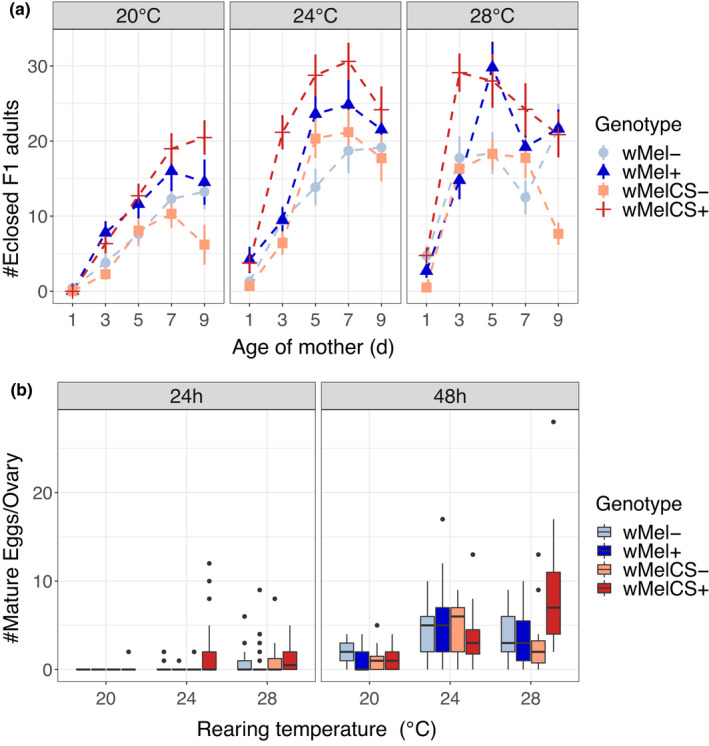
Mean fecundity of *D*. *melanogaster* from Portugal naturally infected with *w*Mel+ (blue) and *w*MelCS+ (red) *Wolbachia* variants and reared at 20, 24 and 28°C compared to counterparts treated with antibiotics (*w*Mel−, light blue and *w*MelCS−, light red). (a) Number of eclosed adult flies laid by females at different ages (1–9 days) reared at three temperatures. The error bars show standard errors. Total *n* = 1593. (b) Mean number of mature eggs per ovariole of females 24 and 48 h after eclosion. Total *n* = 588

We measured female fecundity as the number of eclosed adult offspring per female in 24‐h intervals at different maintenance temperatures. Since Female IDs were not fully tracked during the experiment, we analysed each time point separately to avoid pseudoreplication, which may result in an inflated type‐II error (i.e. false rejection of the null hypothesis). At all four time points (aged 3–9 days), *temperature* and *infection* had highly significant effects on fecundity (Table 4; Figure 4a). *Wolbachia infection* significantly increased offspring number (Table 4; Figure 4a) across all temperatures. The mean number of progeny produced per female was highly temperature‐dependent and lowest at 20°C, where females had on average nine offspring per day, whereas females produced on average 16 and 17 daily offspring at 24 and 28°C, respectively (Table 4, Figure 4a). We further found significant interactions between *infection* and *cross* on day 3 (*F*_1/329_ = 9.6, *p* = 0.002; Table 4) and day 9 (*F*_1/247_ = 12, *p* = 0.001; Table 4). Particularly on day 3, *w*MelCS‐infected females produced more offspring at 24 and 28°C than both uninfected females and females infected with *w*Mel (Tukey HSD; *p* < 0.001 for all comparisons), indicating that *Wolbachia* infections have a positive effect on fecundity and that this effect differs for *Wolbachia* variants. Since such a significant interaction was not found at days 5 and 7, we speculate that *w*MelCS may stimulate an early onset of oogenesis. On day 9, in contrast, wMelCS+ fecundity did only differ from wMel+ and *w*Mel− at 20°C (Tukey HSD; *p* < 0.001). However, *w*MelCS‐infected flies were nevertheless more fecund than uninfected flies (*w*MelCS−) of the same crossing direction, irrespective of temperature (Tukey HSD; *p* ≤ 0.0001 for all comparisons). In addition, peak fecundity of *w*MelCS + females appears to be strongly temperature‐dependent (Figure 4a): while peak fecundity was not reached within 9 days at 20°C, it averaged 7 days at 24°C and 3 days at 28°C.

**TABLE 4:** Type‐III ANOVA *F*‐tests with Satterthwaite's method to approximate degrees of freedom testing for significant effects of *temperature*, *Wolbachia* variant (i.e. direction of *cross*) and *infection* status on female fecundity (measured as number of adult offspring that emerged from eggs that were laid in 24‐h intervals from single females) in four data sets collected at consecutive time points

Effect	3 Days old			5 Days old			7 Days old			9 Days old		
	*F*	*df*	*p*‐Value	*F*	*df*	*p*‐Value	*F*	*df*	*p*‐Value	*F*	*df*	*p*‐Value
**Temp**	**54.4**	**2/329**	**3.7E−21**	**34.3**	**2/320**	**3.3E−14**	**10.9**	**2/296**	**2.8E−05**	**5.9**	**2/247**	**3.1E−03**
Cross	0.2	1/329	0.692	1.5	1/320	0.215	2.8	1/296	0.095	4.6	1/247	0.032
**Infection**	**22.8**	**1/329**	**2.7E−06**	**25.9**	**1/320**	**6.1E−07**	**17.8**	**1/296**	**3.3E−05**	**17.0**	**1/247**	**5.0E−05**
Temp:Cross	2.2	2/329	0.115	1.6	2/320	0.198	0.8	2/296	0.454	2.9	2/247	0.057
Temp:Infection	3.2	2/329	0.043	0.0	2/320	0.996	0.1	2/296	0.884	1.3	2/247	0.277
Cross:Infection	**9.6**	**1/329**	**0.002**	0.2	1/320	0.696	0.3	1/296	0.567	**12.0**	**1/247**	**0.001**
Temp:Cross:Infection	1.0	2/329	0.383	0.1	2/320	0.879	0.5	2/296	0.610	1.9	2/247	0.762

Note

Significant results are highlighted in bold. Note that the significance threshold was Bonferroni‐corrected (α' = 0.05/4 = 0.0125) to account for multiple testing since the data sets are not independent across time points.

To test the hypothesis that *w*MelCS infections stimulate an early onset of oogenesis, we counted the number of mature eggs in ovaries of young females 24 h and 48 h after eclosion. Only 52 (18%) 24‐h‐old females had produced at least one mature egg. Due to excessive zero inflation, we did not statistically analyse this data subset. However, we noted that most of the females with mature eggs (33) were infected with *w*MelCS. For flies aged 48 h, we found a highly significant increase in number of mature eggs with *temperature* (*χ* ^2^ = 59.3, *p* < 1.3e‐13; Table 5). In addition, we found significant interactions between *temperature* and *infection* (*χ* ^2^ = 12.3, *p* = 0.002; Table 5); *cross* and *infection* (*χ* ^2^ = 8.0, *p* = 0.005); and between *temperature*, *cross* and *infection* (*χ* ^2^ = 10.9, *p* = 0.004; Table 5). These results suggest that the onset of oogenesis in *w*MelCS‐infected females begins earlier than in uninfected flies and flies infected with *w*Mel, and that this effect is particularly pronounced at higher temperatures.

**TABLE 5:** Analysis of deviance for the number of mature eggs laid per female of different *cross* and *infection* status reared at different *temperatures* 48 h after eclosion

Effect	*χ*2	*df*	*p*‐Value
**Temp**	**59.3**	**2**	**1.3E−13**
Cross	0.1	1	0.792
Infection	0.1	1	0.752
Temp:Cross	1.6	2	0.444
**Temp:Infection**		**2**	**0.002**
**Cross:Infection**	**8.0**	**1**	**0.005**
**Temp:Cross:Infection**	**10.9**	**2**	**0.004**

Note

Significant results are highlighted in bold.

### 3.5 Longevity

**FIGURE 5:**
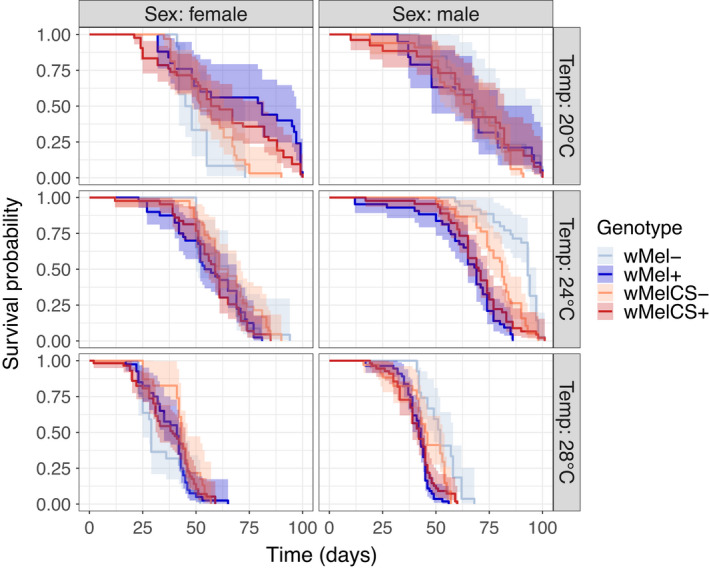
Longevity of *D*. *melanogaster* naturally infected with *w*Mel+ (dark blue) and *w*MelCS+ (dark red) *Wolbachia* reared at 20, 24 and 28°C compared to flies treated with antibiotics (*w*Mel− and *w*MelCS−, light colours). The shaded polygons indicate 95% confidence intervals. Total *n* = 824

A well‐described trade‐off in life‐history evolution is decreased longevity in case of higher early fecundity (cost of reproduction), which is commonly explained by allocation trade‐offs due to limited resources (Flatt, 2011). We found highly significant effects of *temperature* and *sex* on longevity (*temperature*; *χ* ^2^ = 419.8, *p* < 2.2e‐16; *sex*; *χ* ^2^ = 78.1, *p* < 2.2e‐16; Table 6), but also significant two‐way interactions between *sex* and *infection* (*χ* ^2^ = 35.8, *p* = 2.2e‐09; Table 6) and *temperature* and *infection* (*χ* ^2^ = 46.4, *p* < 8.4e‐11; Table 6). The significant interaction between *sex* and *cross* (*χ* ^2^ = 6.3, *P* 0.012; Table 6) indicates sex‐linked effects with respect to the direction of the *cross* but independently of *Wolbachia* infections. Additionally, we observed highly significant three‐way interactions between *sex*, *cross* and *infection* (*χ* ^2^ = 22.6, *p* < 2.0e‐06; Table 6) and *sex*, *temp* and *infection* (*χ* ^2^ = 9.5, *p* = 0.009; Table 6), indicating that effects on lifespan of *Wolbachia infection* differ with temperature and that differences among *Wolbachia* variants are sex‐specific (as above). Males infected with *w*Mel reared at 24 and 28°C died sooner than uninfected males (pairwise Tukey HSD post hoc test, *p* < 0.0001 and *p* = 0.008, respectively; Figure 5). By contrast, both *w*Mel and *w*MelCS females reared at 20°C lived longer than uninfected females (pairwise Tukey HSD post hoc test, *p* < 0.0001 and *p* = 0.02, respectively; Figure 5, Table 6).

**TABLE 6:** Analysis of deviance for lifespan in *D*. *melanogaster* flies infected with different *Wolbachia* variants and reared at different temperatures

Effect	*χ*2	*df*	*p*‐Value
**Sex**	**78.1**	**1**	**<2.2E‐16**
**Temp**	**419.8**	**2**	**<2.2E‐16**
Cross	0.1	1	0.763
Infection	0.6	1	0.457
**Sex:Temp**	**7.3**	**2**	**0.026**
**Sex:Cross**	**6.3**	**1**	**0.012**
Temp:Cross	2.9	2	0.233
**Sex:Infection**	**35.8**	**1**	**2.2E‐09**
**Temp:Infection**	**46.4**	**2**	**8.4E‐11**
Cross:Infection	0.0	1	0.959
Sex:Temp:Cross	1.0	2	0.621
**Sex:Temp:Infection**	**9.5**	**2**	**0.009**
**Sex:Cross:Infection**	**22.6**	**1**	**2.0E‐06**
Temp:Cross:Infection	3.4	2	0.185
Sex:Temp:Cross:Infection	0.5	2	0.791

Note

Significant results are highlighted in bold

## 4 DISCUSSION

Systematic studies of *D*. *melanogaster* long‐term lab strains and worldwide populations have uncovered a recent global turnover of two *Wolbachia* variants resulting in the replacement of *w*MelCS by *w*Mel within half a century (Riegler et al., 2005). However, it is still unknown which mechanisms underlie such rapid evolutionary change. This motivated us to disentangle the fitness consequences of these two *Wolbachia* variants on natural *D*. *melanogaster* populations while taking into account temperature, which is one of the most important environmental factors influencing the physiology and life history of all organisms (Angilletta et al., 2004; Thomas & Blanford, 2003). Temperature further affects the interaction dynamics between host and microbe (reviewed in Corbin et al., 2017). Moderate temperatures between 22 and 26°C are usually considered comfortable for both partners of the *Wolbachia*–*Drosophila* association (Gora et al., 2020; Hague et al., 2020; López‐Madrigal & Duarte, 2019). In general, high temperatures lead to depletion of bacteria from the host, while lower temperatures slow down the replication of the symbiont and alleviate potential fitness costs (Chrostek et al., 2021; Hague et al., 2020; Strunov et al., 2013), although there are exceptions (Min & Benzer, 1997; Mouton et al., 2005). In obligate mutualistic interactions, like *w*Pau in neotropical *D*. *paulistorum* hosts, increased temperatures deplete the mutualistic endosymbiont and consequently reduce host fitness and fecundity significantly (Ehrman & Powell, 1982; Miller et al., 2010; Schneider et al., 2019). Thus, temperature might serve as a key regulator of host–microbe interactions. To investigate this hypothesis, we analysed the impact of temperature on various fitness‐related traits in juvenile and adult wild‐caught *D*. *melanogaster* flies infected with two different natural *Wolbachia* variants, as summarized in Table 7. To disentangle host phenotypic differences due to *Wolbachia* infections and/or due to differences in the host genetic background, we carried out all phenotypic assays in F1 hybrids from reciprocal crosses among the pure host strains. Importantly, except for a significant interaction between *sex* and *cross* in the longevity data set, which may indicate sex‐specific differences with respect to the direction of the cross, we did not observe significant effects of the main factor *cross* in any of our experiments. This suggests that our experimental design successfully removed potentially confounding phenotypic differences caused by the host genetic background. Conversely, we found significant interactions between the factors *cross* and *infection* and higher order interactions including these two factors, which indicates that the two investigated *Wolbachia* variants influence several fitness‐related host phenotypes differently.

**TABLE 7:** Variable effects of *w*Mel versus *w*MelCS *Wolbachia* infection on various life‐history traits of *D*. *melanogaster* reared at three different temperatures

Trait	20°C	24°C	28°C
Development time	No effect	No effect	No effect
Body size	No effect	No effect	No effect
Fecundity	*w*MelCS+ > *w*MelCS−	*w*MelCS+ > *w*MelCS− *w*MelCS+ > *w*Mel+	*w*MelCS+ > *w*MelCS−
Oogenesis	No effect	*w*MelCS+ > *w*Mel+	*w*MelCS+ > *w*MelCS− *w*MelCS+ > *w*Mel+
Longevity females	*w*MelCS+ > *w*MelCS− *w*Mel+ > *w*Mel−	No effect	No effect
Longevity males	No effect	*w*Mel+ < *w*Mel−	*w*Mel+ < *w*Mel−

### 4.1 Developmental life‐history traits are not influenced by *Wolbachia* infections

Consistent with earlier reports from many species, temperature had a major impact on development time and body size of *D*. *melanogaster* (see Flatt, 2020 for a comprehensive review). By contrast, there were no direct effects of *Wolbachia* infection nor variant, nor interactions with temperature on juvenile development time and resulting adult body size, which is also in line with a previous study (Harcombe & Hoffmann, 2004). It is known that environmental conditions and physiological interactions with an endosymbiont during development may directly affect the resulting adult phenotype (Grenier & Leulier, 2020). For instance, rearing *D*. *simulans* larvae infected with *Wolbachia* at high temperatures increased cytoplasmic incompatibility in males (Clancy & Hoffmann, 1998), which is beneficial for the spread of *Wolbachia* (Turelli & Hoffmann, 1995). The absence of effects observed here in the juvenile life stages of *D*. *melanogaster* hosts might therefore be the result of overall low *Wolbachia* titre (infection) levels of juveniles (Stevanovic et al., 2015; Strunov et al., 2013). In line with this hypothesis, a previous study showed that *w*Mel‐infected larvae with low titre levels did not exhibit any resistance to the *Drosophila* C virus, (DCV; Stevanovic et al., 2015), possibly due to subthreshold physiological effects induced by the endosymbiont on the host. This interpretation is supported by a recent study of Chrostek et al. (2021), who found that infected individuals reared at 18°C did not exhibit enhanced resistance against DCV, contrary to those reared at 25°C. Low‐rearing temperatures thus seem to suppress replication of *Wolbachia* during development (Hague et al., 2020), and might subsequently influence the life history of the adult in terms of fecundity and lifespan.

Alternatively, the absence of *Wolbachia* effects on developmental life‐history traits might be explained by differential activity of the endosymbiont during this early period of the host life cycle. A comprehensive analysis of *Wolbachia* gene expression across the *D*. *melanogaster* life cycle shows that the bacteria have significantly distinctive expression patterns in early larvae, late pupae and adults (Gutzwiller et al., 2015). A follow‐up study that reanalysed previously published *Wolbachia* RNA‐Seq transcriptomic data uncovered that *w*Mel variant genes which affect ribosome biosynthesis and translation of the host are consistently upregulated during early life relative to adult stages (Chung et al., 2020). In this context however, we did not observe any effects of *Wolbachia* on developmental life‐history traits at any temperature under laboratory conditions, possibly due to overall low titre and/or possible physiological dormancy of bacteria during the larval stage of development. Further experimental work focusing on titre levels and physiological effects of *Wolbachia* at different developmental stages of the host is needed to test if the density of infection influences the extent of phenotypic effects at different developmental stages of the host.

### 4.2 Adult life‐history traits are affected by *Wolbachia* variant in interaction with temperature

In contrast to developmental traits, *Wolbachia* considerably affected adult fitness components in our experiments. The effects of *Wolbachia* variants on host life‐history traits were environment dependent and differed between host genetic backgrounds. Our results are in line with recent findings in natural *D*. *melanogaster* populations from Uman, Ukraine. Serga et al. (2021) showed that *Wolbachia* positively influenced reproduction in only some *Drosophila* genotypes, at a cost of reduced lifespan and lower stress resistance. According to Chrostek et al. (2013), *w*MelCS‐infected flies show higher bacterial titre than those infected by *w*Mel, which may result in decreased longevity despite higher protection against RNA viruses. Similar patterns were observed in *D*. *simulans*, which has been artificially transfected with various non‐native *Wolbachia* variants from other *Drosophila* species (Martinez et al., 2014, 2015), showing that high titre infections often result in negative fitness effects such as reduced fecundity, egg hatching rate or male fertility. By contrast, our study showed positive effects of *w*MelCS, and to a lesser extent also of *w*Mel, on fecundity and longevity in *D*. *melanogaster* populations from Portugal. Thus, our findings are not consistent with previous data (Chrostek et al., 2013), which might be explained by differences among the fly strains investigated. Chrostek et al. (2013) and Martinez et al. (2014, 2015) used long‐term and highly inbred *D*. *melanogaster* or *D*. *simulans* lab strains artificially transfected or introgressed with various native or alien *Wolbachia* variants. Due to low effective population sizes, these *Drosophila* hosts may have accumulated novel mutations with negative effects for life‐history traits (Charlesworth & Charlesworth, 1987). In contrast, our study used recently collected wild flies harbouring their native *Wolbachia* endosymbionts, and in addition, we generated F1 hybrid offspring to eliminate negative inbreeding effects on host fitness. However, it remains unclear to which extent these *Wolbachia*‐induced effects reflect general patterns of *Wolbachia*–*Drosophila* interactions or instead represent artefacts from non‐native infections with *Wolbachia* variants that are not specific to their hosts.

The only negative effect associated with *Wolbachia* infections found in our study was observed at higher temperatures (24 and 28°C) in males infected with *w*Mel, which died sooner than uninfected males. In line with our results, Fry and Rand (2002) similarly found that the influence of *Wolbachia* on survival differs for the sexes. Since *Wolbachia* is maternally transmitted to enhance its own transmission, it is advantageous for the endosymbiont to have a positive effect on the survival of infected females but not necessarily on that of males (Werren et al., 2008). Interestingly, the negative effect on longevity was not observed in *w*MelCS‐infected males. The variant *w*MelCS is considered the ancestral infection type of *D*. *melanogaster*, which was more recently replaced by *w*Mel globally (Early & Clark, 2013; Ilinsky, 2013; Richardson et al., 2012; Riegler et al., 2005). Thus, potential negative effects of *w*MelCS on longevity of wild‐caught hosts might have therefore attenuated considerably with time because of longer host–symbiont co‐evolution.

In addition, *w*MelCS infections have been shown to cause a shift in thermal preference towards cooler temperatures in *D*. *melanogaster* hosts, but not in *w*Mel‐infected flies (Arnold et al., 2019; Truitt et al., 2019). Lower temperatures prolong development time of the host and hence augment generation times. Thus, thermal preferences for lower temperatures may decrease the fitness of *w*MelCS‐infected flies in ephemeral environments such as rapidly decaying fruit. However, a recent study of thermal preferences in various *Drosophila* species infected with different *Wolbachia* variants failed to find a similar behavioural effect specific to *w*Mel and *w*MelCS in other *D*. *melanogaster* host backgrounds of long‐term lab strains (Hague et al., 2020). Overall, these studies suggest considerable variation in fitness impact of natural and artificial *Wolbachia* infections. According to our data, *w*MelCS‐infected flies performed better at higher temperatures than flies with *w*Mel infections, which contradicts the results of thermal preference experiments using introgressed and highly inbred lab strains (Arnold et al., 2019; Truitt et al., 2019).

Besides the global effects of *Wolbachia* on fly life history, there are differences between bacterial strains in terms of intracellular communication with the host that are still largely unknown (Strunov et al., 2022). A recent publication shows how the closely related *w*Mel and *w*MelCS variants are differently regulated by the autophagy machinery of the hub cells in *D*. *melanogaster* testes, as only *w*MelCS *Wolbachia* are able to escape the elimination by autolysosomes (Deehan et al., 2021). As shown earlier, *Wolbachia* can influence insulin signalling in *D*. *melanogaster* (Ikeya et al., 2009), which could result in various effects of *Wolbachia* infections on fecundity and lifespan, depending on the strain of bacteria.

One of the most striking findings of our study is the earlier peak fecundity at 28°C of *w*MelCS‐infected females, in combination with their overall higher fecundity at all temperatures. The positive effect of *Wolbachia* on fecundity was shown previously in *D*. *simulans* and *D*. *mauritiana*, where the *D*. *mauritania*‐specific *Wolbachia* variant *w*Mau increased the mitotic activity of germline stem cells (GSCs) and decreased the programmed cell death in the germarium (Fast et al., 2011). However, in a more recent study, this finding could not be reproduced (Meany et al., 2019). Increased fecundity through GSCs manipulation by *Wolbachia* was reported in another insect, the hemipteran *Laodelphax striatellus* (Guo et al., 2020), suggests the existence of a common mechanism of endosymbiont interference promoting female host reproduction. Thus, it is possible that the *w*MelCS variant acts similarly in wild‐caught *D*. *melanogaster*. Enhanced reproduction is costly and might lead to a reduced lifespan of the female due to energetic trade‐offs (Flatt, 2011). However, we did not observe any additional costs of *w*MelCS infection for females in our study, which might have remained undetected under laboratory conditions of sufficient food supply and controlled temperature, or may manifest in traits that were not investigated here. In nature, the cost of producing a higher number of progeny might lead to otherwise reduced performance of *w*MelCS‐infected flies, and eventually to replacement by a variant such as *w*Mel with milder effects. Further studies investigating *Wolbachia*‐borne trade‐offs under natural conditions are necessary to test this hypothesis (e.g. see Utarini et al., 2021).

Besides phenotypic variation induced by different *Wolbachia* types, several current studies uncovered ample genetic variation within *Wolbachia* variants, which might lead to differences in host–symbiont interactions even if the host is infected with the same variant (Richardson et al., 2012; Scholz et al., 2020). Hague et al. (2022), for example, recently described a single SNP in the outer membrane protein (WspB) of *w*Mel that might be a candidate for thermal sensitivity in bacteria. Another recent work by Gu et al. (2022) reported the emergence of a new *w*MelM strain of *Wolbachia* in *Aedes aegypti* that induces increased heat tolerance in comparison to a *w*Mel variant from an Australian *D*. *melanogaster* population which differs in 36 SNPs and small indels. Future studies to quantitatively link phenotypic effects and genomic variation of host and symbiont are needed to better understand mechanistically how host–symbiont interactions influence fitness.

## 5 CONCLUSION

We observed no influence of bacterial infection on developmental life‐history traits. However, both *Wolbachia* variants increased lifespan and fecundity of host females depending on the thermal environment without apparent fitness trade‐offs. Interestingly, the ancestral *Wolbachia* variant *w*MelCS had a positive effect on host fitness components compared to uninfected and *w*Mel‐infected flies. Therefore, our study cannot provide a conclusive explanation of the recent global replacement of *w*MelCS by *w*Mel. Together with other recent studies, it however suggests varying degrees of reproductive costs induced by *Wolbachia* under variable natural environmental conditions, which should ultimately affect the evolutionary fate of the two major *Wolbachia* variants in nature.

Comparisons with previous studies reveal differences in fitness effects of *Wolbachia* between long‐term laboratory and wild‐caught *Drosophila* hosts. Long‐term evolution of hosts and endosymbionts in the lab likely influences interactions among host genotype, symbiont genotype and the environment (G × G × E), and emphasizes the importance of studying host–microbe interplay in nature. Such data are needed to further investigate the effect of environmental conditions and genetic variation of hosts and symbionts in the context of ongoing applications of *Wolbachia* in biocontrol to fight the spread of viral diseases by mosquito vectors in natural populations (Utarini et al., 2021).

## AUTHORS CONTRIBUTIONS

Anton Strunov involved in conceptualization, formal analysis, validation, writing—original draft; Sina Lerch involved in conceptualization, investigation, formal analysis, data curation, visualization, writing—review & editing; Wolf Blanckenhorn involved in writing—review & editing, statistical advice, supervision, funding acquisition; Wolfgang Miller involved in conceptualization, writing—review & editing, supervision; Martin Kapun involved in conceptualization, formal analysis, visualization, supervision, funding acquisition, writing—review & editing, project administration.

### OPEN RESEARCH BADGES

This article has earned an Open Data Badge for making publicly available the digitally‐shareable data necessary to reproduce the reported results. The data is available at https://doi.org/10.5061/dryad.sxksn035v.

### PEER REVIEW

The peer review history for this article is available at https://publons.com/publon/10.1111/jeb.14016.

## Supporting information

Supplementary Material

## Data Availability

All raw data are deposited at Data Dryad under https://doi.org/10.5061/dryad.sxksn035v or as supplementary material.
