# Supplementary material for: Complex effects of environment and Wolbachia infections on the life history of Drosophila melanogaster hosts
Source: J Evol Biol. 2022 May 9;35(6):788–802. doi: 10.1111/jeb.14016 (PMC9321091; doi:10.1111/jeb.14016)
Supplement: Supplementary file 1 — Supplementary Material [file JEB-35-788-s001.zip › Supplemental data/Figures/MatureEggs.pdf]

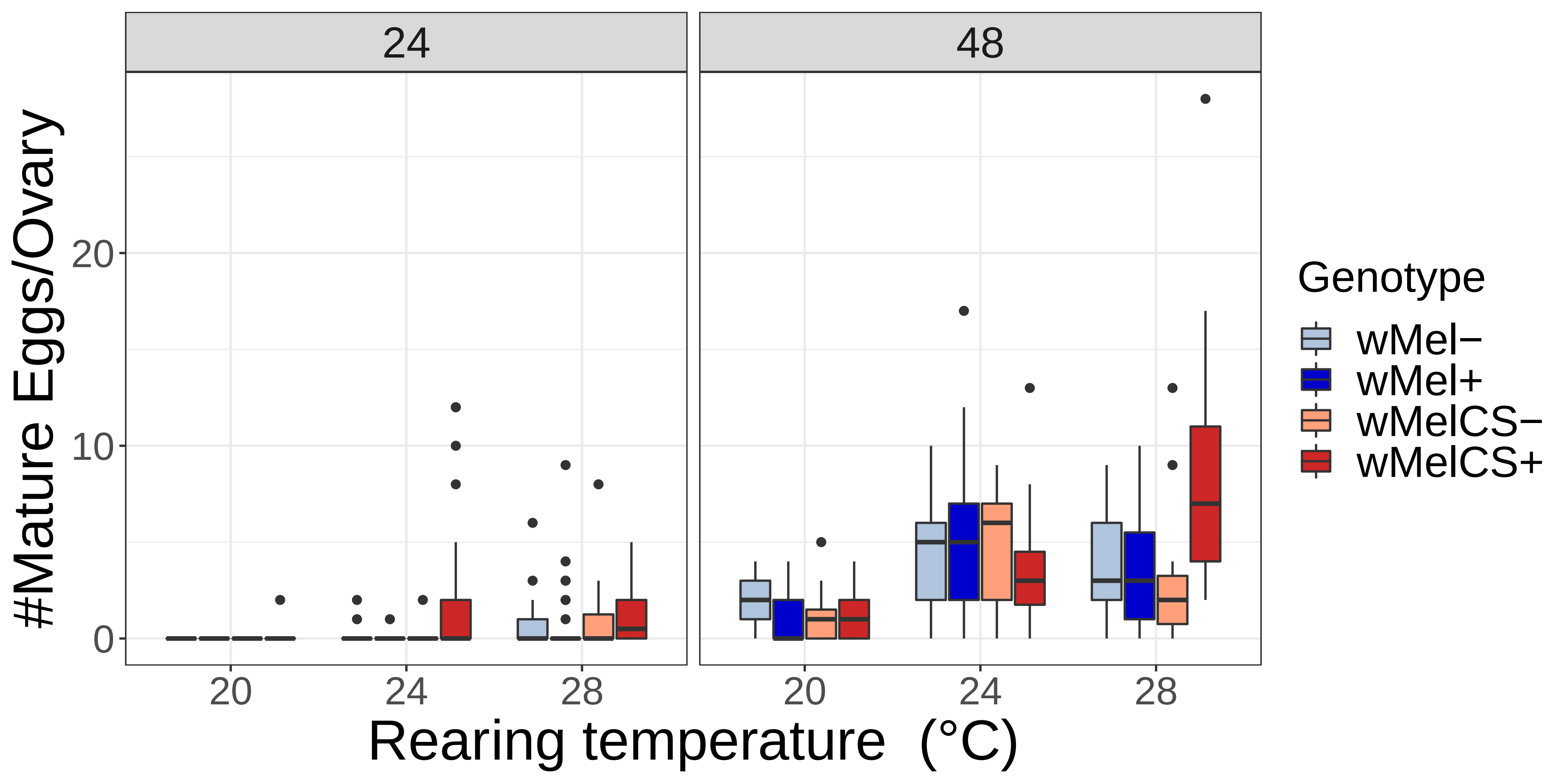
24
48
#Mature Eggs/Ovary
0
10
20
20
24
28
20
24
28
Rearing temperature (°C)
Genotype
wMel−
wMel+
wMelCS−
wMelCS+
