# Supplementary figures and images for: Complex effects of environment and Wolbachia infections on the life history of Drosophila melanogaster hosts
Source: J Evol Biol. 2022 May 9;35(6):788–802. doi: 10.1111/jeb.14016 (PMC9321091; doi:10.1111/jeb.14016)

Development time (h)

female

male

Genotype

- wMel-
- wMel+
- wMelCS-
- wMelCS+

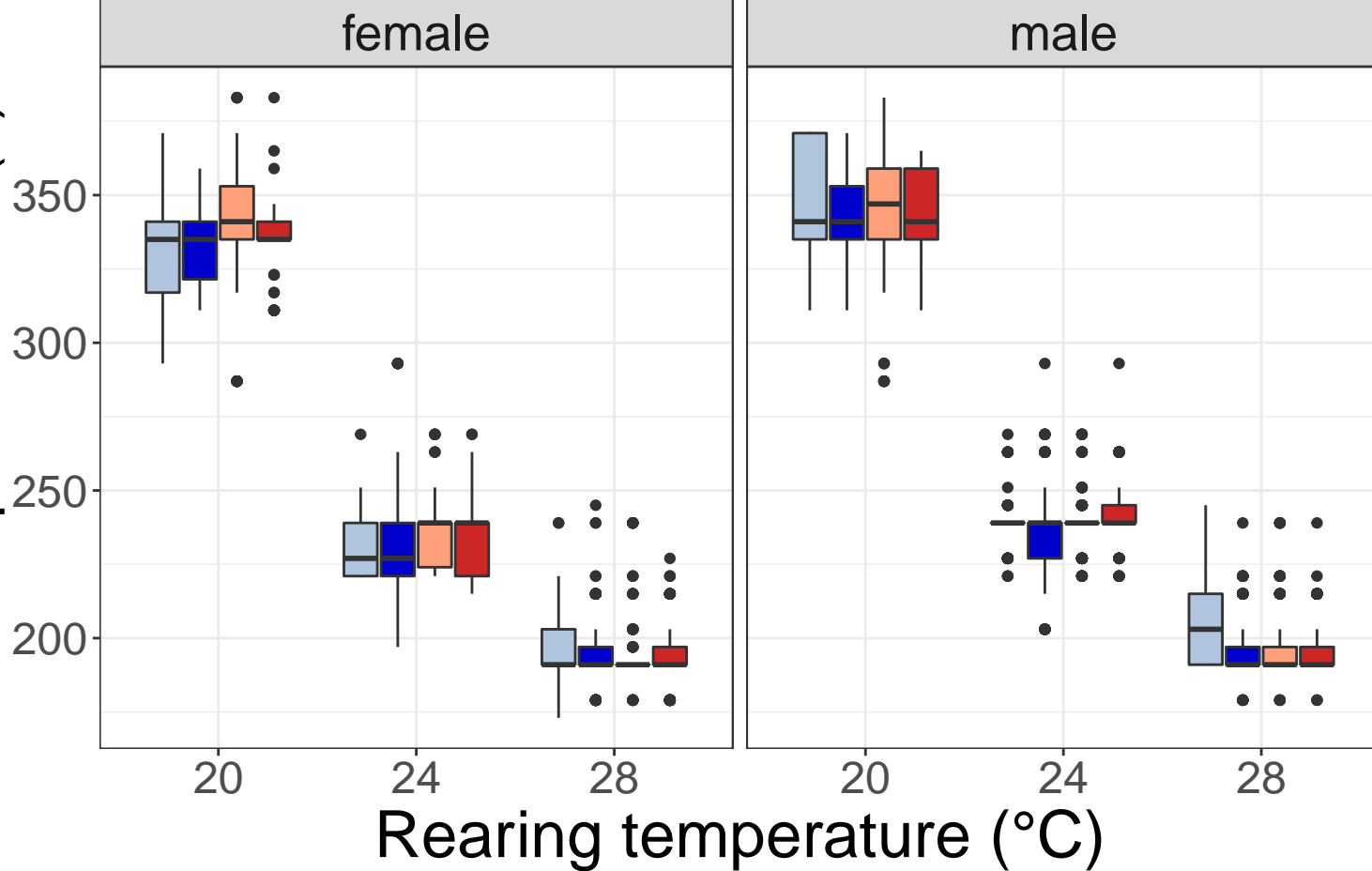

Supplement: Supplementary file 1 — Supplementary Material [file JEB-35-788-s001.zip › Supplemental data/Figures/Developmenttime.pdf]

#Enclosed F1 adults

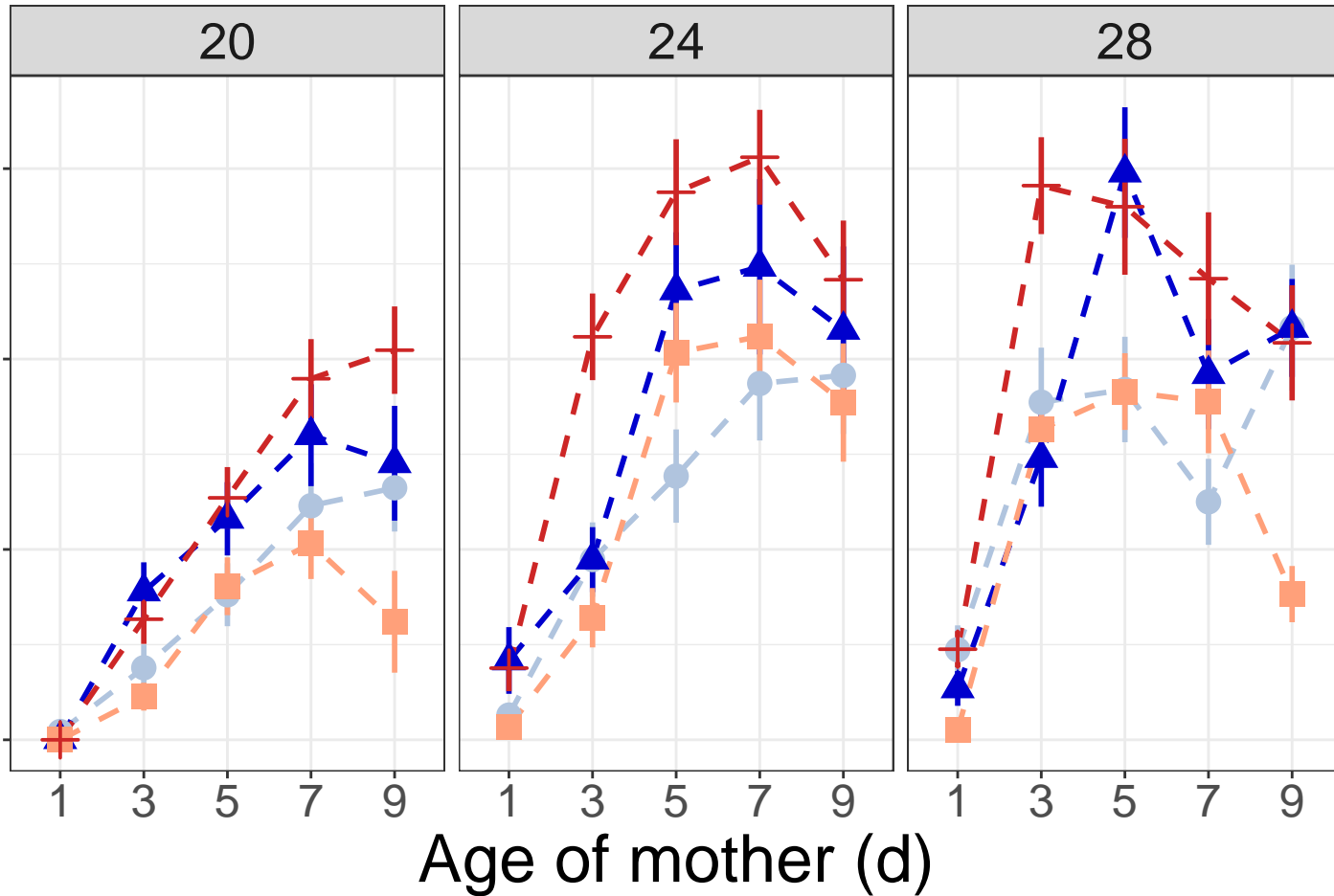

Genotype

- wMel-
- wMel+
- wMelCS-
- wMelCS+

Supplement: Supplementary file 1 — Supplementary Material [file JEB-35-788-s001.zip › Supplemental data/Figures/Fecundity.pdf]

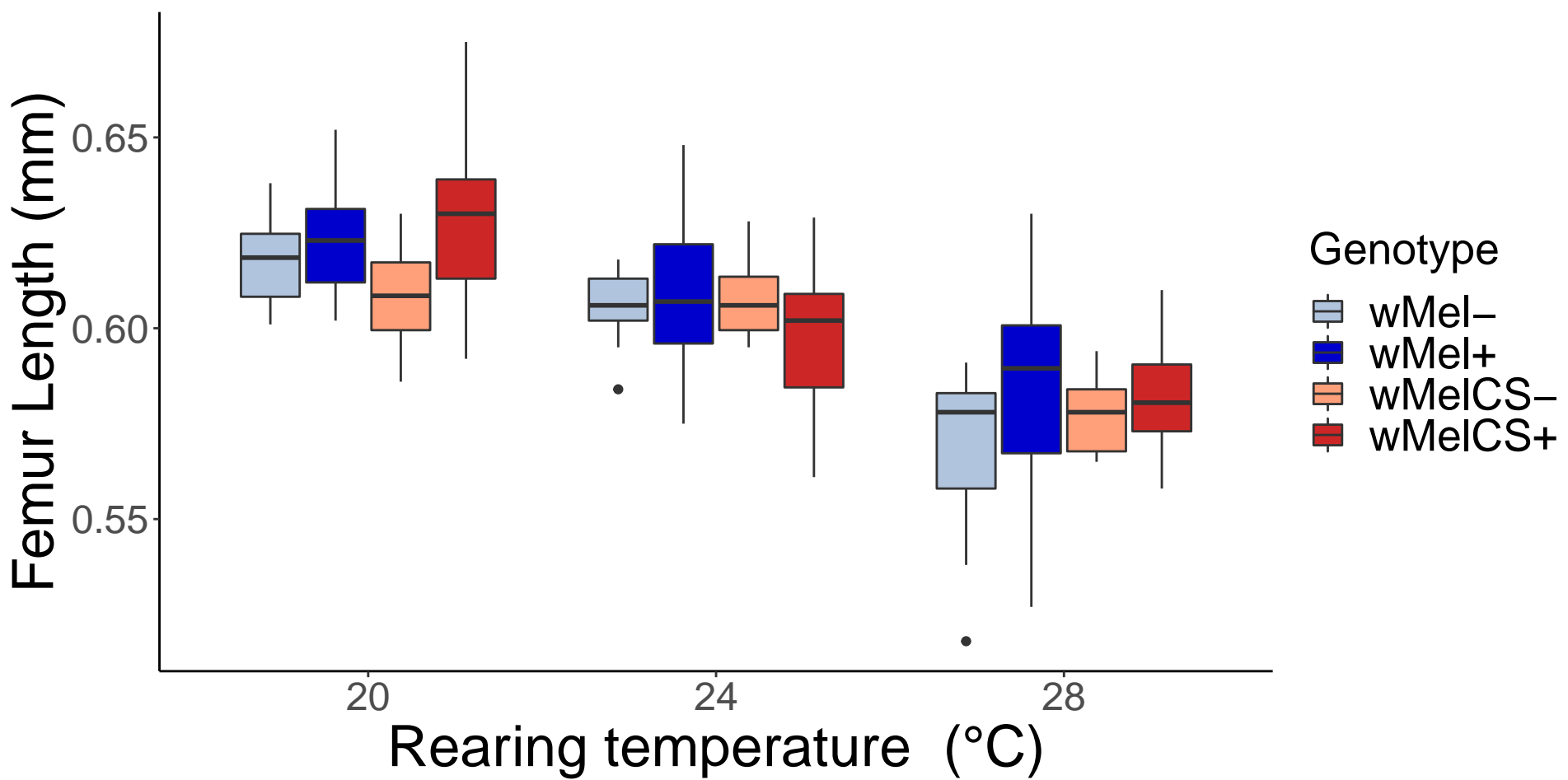

Supplement: Supplementary file 1 — Supplementary Material [file JEB-35-788-s001.zip › Supplemental data/Figures/FemurLength.pdf]

A

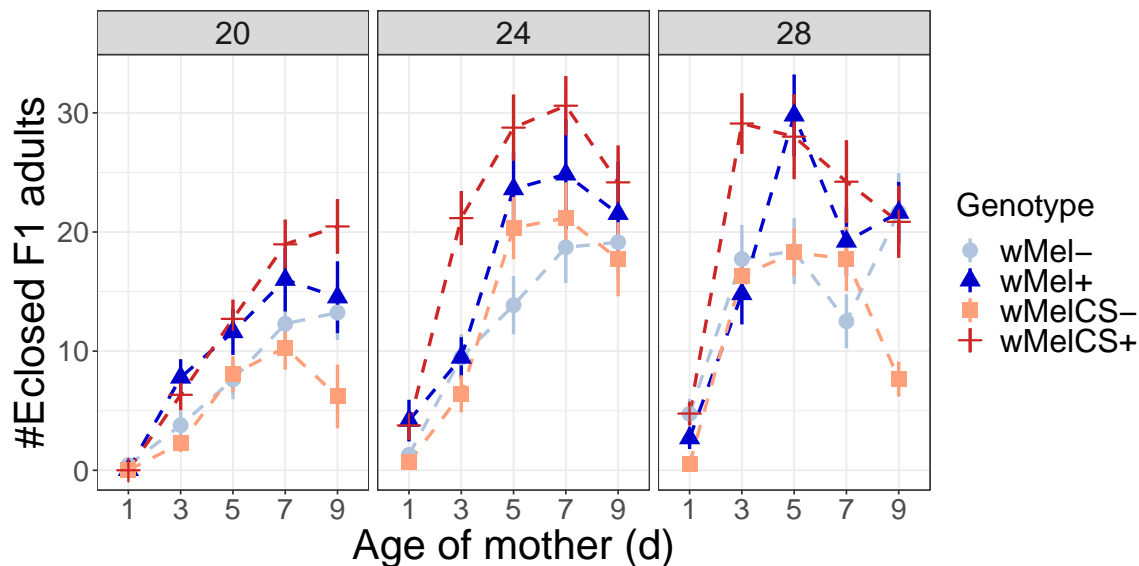

B

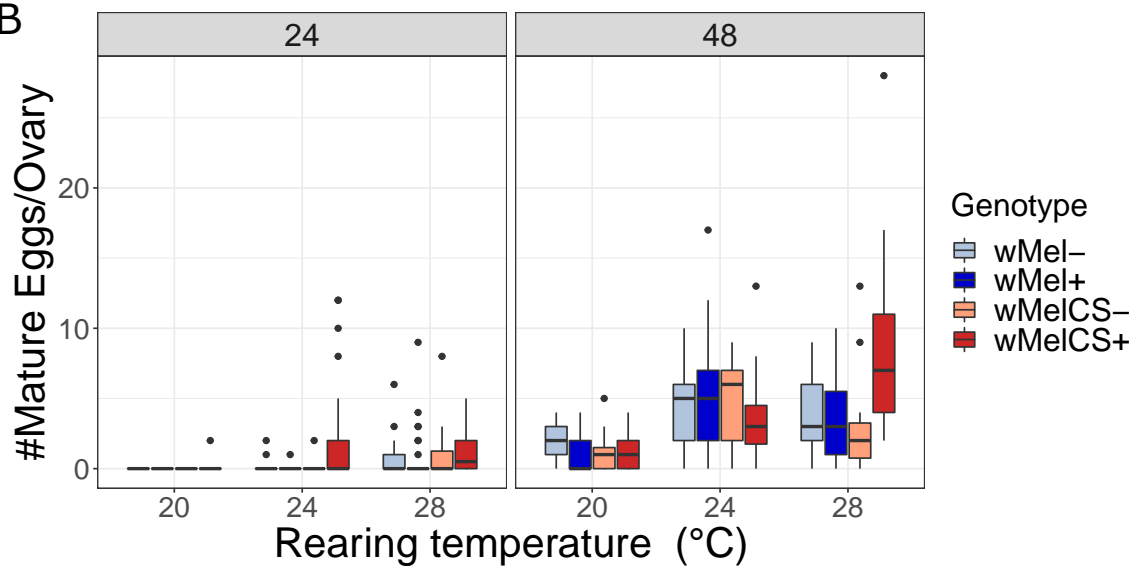

Supplement: Supplementary file 1 — Supplementary Material [file JEB-35-788-s001.zip › Supplemental data/Figures/Figure 4.pdf]

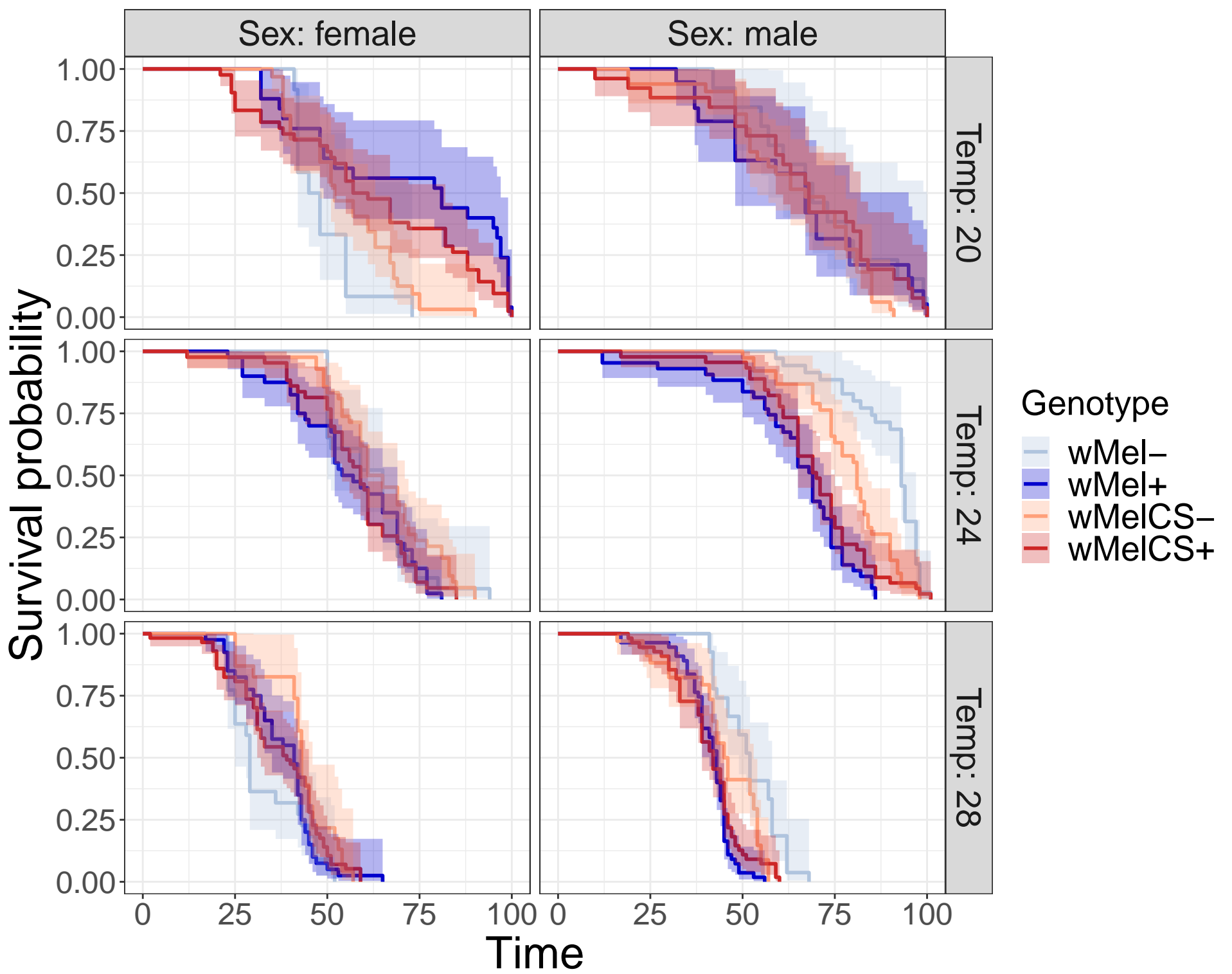

Supplement: Supplementary file 1 — Supplementary Material [file JEB-35-788-s001.zip › Supplemental data/Figures/Longevity.pdf]

#Ovarioles/Ovary

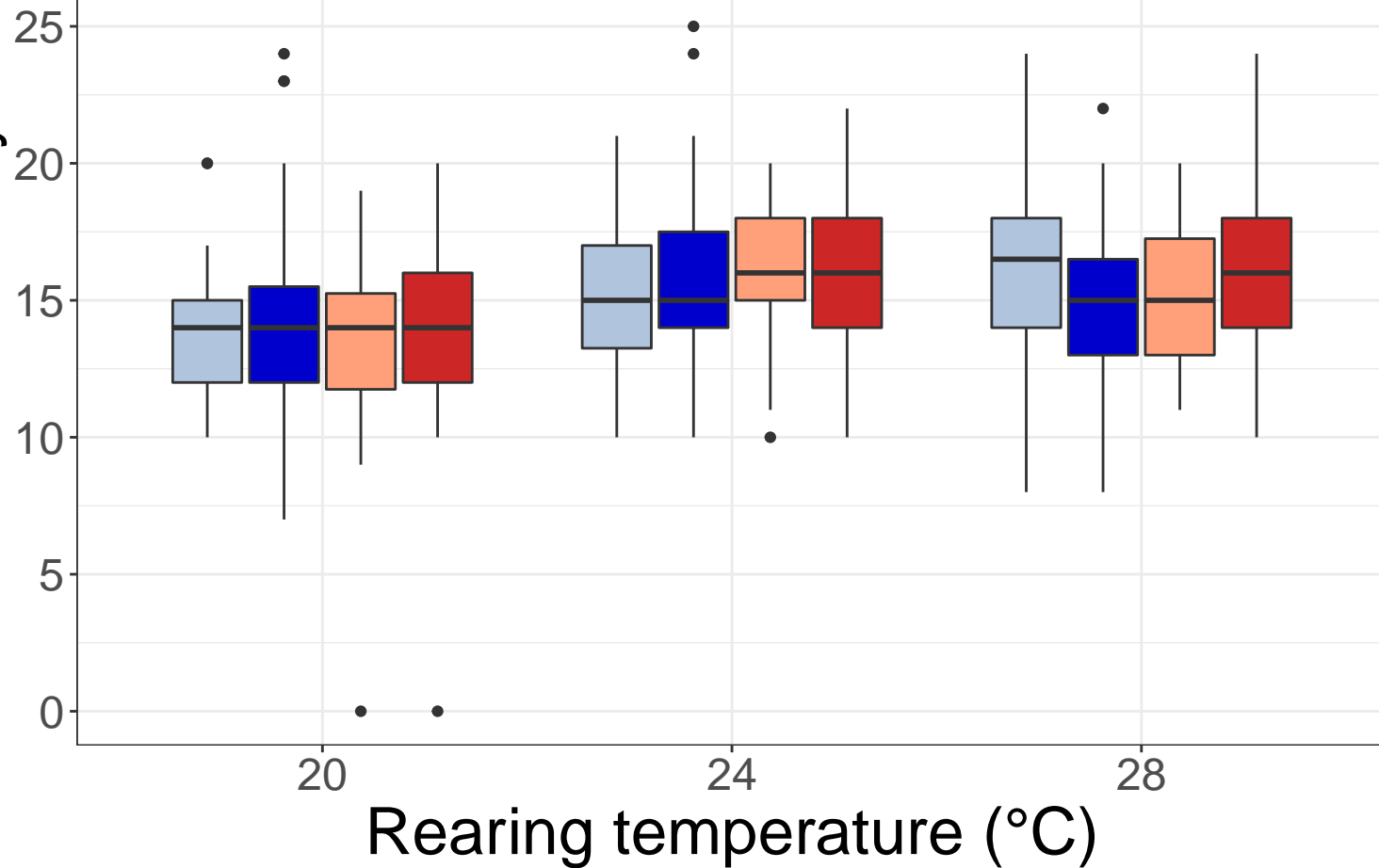

Genotype

- wMel-
- wMel+
- wMelCS-
- wMelCS+

Supplement: Supplementary file 1 — Supplementary Material [file JEB-35-788-s001.zip › Supplemental data/Figures/Ovaries.pdf]

# VNTR-141 PCR

wMel - 1330 bp

wMelCS - 1189 bp

(according to Riegler et al., 2012)

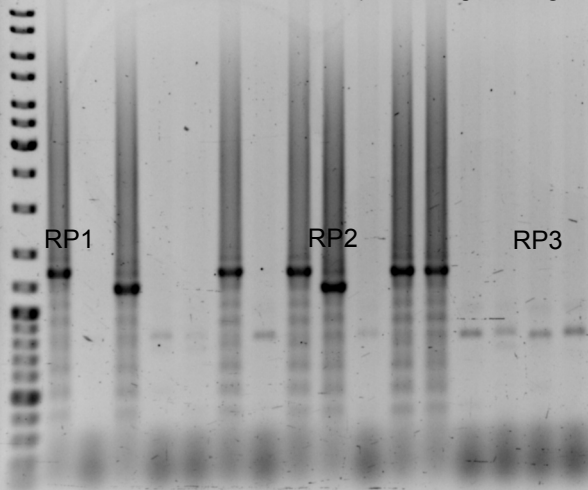

Supplement: Supplementary file 1 — Supplementary Material [file JEB-35-788-s001.zip › Supplemental data/gel pictures/VNTR141-Portugal.pdf]

# WSP PCR

RP (Portugal) 1-16

wMel wMelPop STC- Dummy Blank

1500bp

1000bp

500bp

RP1

RP2

RP3

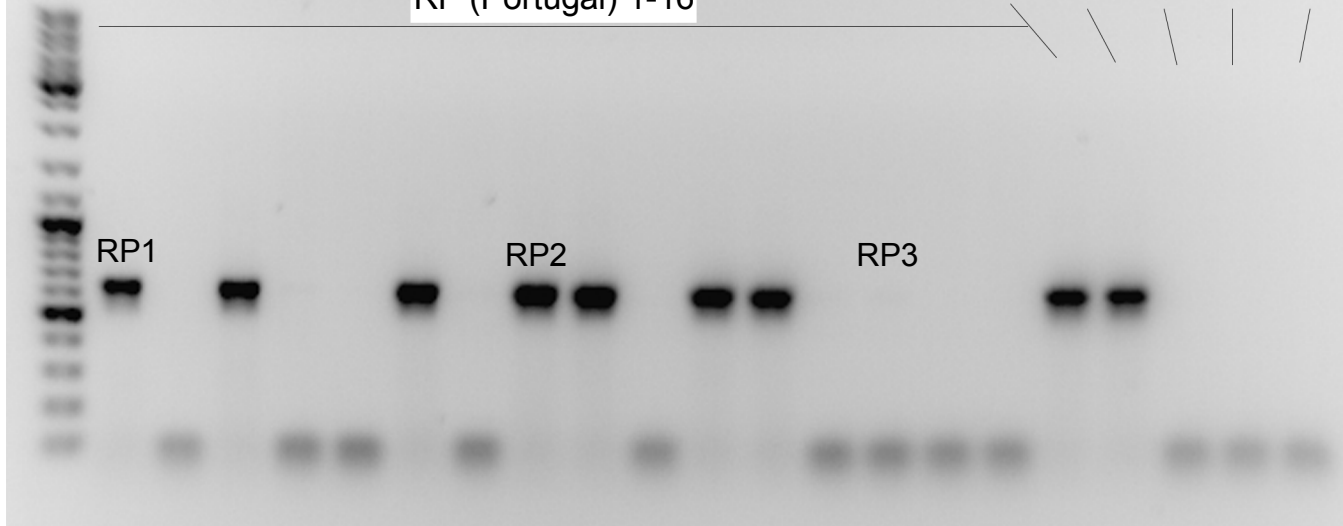

Supplement: Supplementary file 1 — Supplementary Material [file JEB-35-788-s001.zip › Supplemental data/gel pictures/wsp PCR results.pdf]
